# Acute Febrile Illness Surveillance for Estimating Population Immunity, Dominican Republic, 2021

**DOI:** 10.3201/eid3204.251205

**Published:** 2026-04

**Authors:** Eric J. Nilles, Cecilia Then Paulino, Marietta Vasquez, William Duke, Petr Jarolim, Ronald Skewes Ramm, Adam Kucharski, Colleen L. Lau

**Affiliations:** Harvard Humanitarian Initiative, Cambridge, Massachusetts, USA (E.J. Nilles); Brigham and Women's Hospital, Boston, Massachusetts, USA (E.J. Nilles, P. Jarolim); Ministry of Public Health and Social Assistance, Santo Domingo, Dominican Republic (C.T. Paulino, R.S. Ramm); Yale School of Medicine, New Haven, Connecticut, USA (M. Vazquez); National University Pedro Henríquez Ureña, Santo Domingo (W. Duke); Harvard Medical School, Boston (P. Jarolim); London School of Hygiene & Tropical Medicine, London, UK (A. Kucharski); The University of Queensland, Brisbane, Queensland, Australia (C.L. Lau)

**Keywords:** Acute febrile illness, respiratory infections, COVID-19, SARS-CoV-2, viruses, surveillance, serosurvey, population immunity, Dominican Republican

## Abstract

We assessed whether acute febrile illness surveillance could provide timely estimates of population immunity. In the Dominican Republic, antibody levels and inferred protection were similar between surveillance data and household survey serum samples, suggesting that surveillance platforms may offer a scalable approach to track population-level protection.

Cross-sectional population-representative serosurveys currently serve as the standard for estimating population immunity. However, the discrete timeframe, high cost, logistical complexity, and long timelines of that survey method often limit its utility during rapidly evolving outbreaks, when timely public health decision-making is essential. The COVID-19 pandemic evidenced this limitation, when the processing of serologic samples for a national household serosurvey in the Dominican Republic (*1*) in 2021—although methodologically rigorous—failed to keep up with a rapidly shifting postvaccine and postvariant immune landscape.

Researchers have referenced models based on reported case and death data to estimate cumulative infections (*2*), but those approaches rely on strong assumptions about testing access and outcome ascertainment and may be prone to error for pathogens with high proportions of asymptomatic infection. Moreover, such models estimate incidence rather than the level of immune protection in the population, which is the quantity most directly relevant for public health decision-making for pathogens that generate only partial or nonsterilizing immunity. There is a need, therefore, for alternative surveillance approaches, particularly those that can monitor changes over time and are rapid, scalable, low-cost, and feasible during periods of widespread social disruption (*3*).

We evaluated whether routinely collected serologic data from an acute febrile illness (AFI) clinical surveillance platform could serve as a proxy for estimating population immunity, using COVID-19 as proxy. We compared SARS-CoV-2 spike antibody data (Roche Diagnostics, https://www.roche.com) from 2 sources collected during July–October 2021 in the same Dominican Republic provinces: a longitudinal AFI surveillance system embedded in routine healthcare settings ( “surveillance”) (*4*), which included routine blood collection for serologic testing; and a multistage, population-representative household serologic survey (“survey”) (*1*). We matched surveillance participants to survey participants by propensity score 1:5 using age and number of COVID-19 vaccine doses, reflecting a pragmatic approach based on covariates routinely available in surveillance systems. We looked at baseline characteristics before and after matching (Table). To evaluate the potential for type II error (failing to detect a meaningful difference when one exists), we estimated the detectable difference in the proportion above variant-specific protection thresholds given the matched sample sizes (surveillance, n = 115; survey, n = 575), which provided ≥80% power (2-sided α = 0.05) to detect absolute differences of ≈6–8 percentage points. For each variant-specific protection threshold, we estimated the proportion of participants exceeding the threshold within each group using exact binomial methods. We evaluated differences between groups using 2-sample tests for equality of proportions with continuity correction. Those tests evaluate a 2-sided null hypothesis of equal proportions. We used the prespecified +10-percentage-point margin as a benchmark of public health relevance and not as a formal statistical equivalence test (Appendix).

**Table:** Baseline characteristics of surveillance and survey participants before and after propensity score matching for study of acute febrile illness surveillance for estimating population immunity, Dominican Republic, 2021*

Variable	Surveillance, n = 115	Survey, unmatched, n = 962	Survey, matched,† n = 575
Age group, y			
0–14	10 (8.7)	91 (9.5)	55 (9.6)
15–34	52 (45.2)	316 (32.8)	239 (41.6)
35–54	29 (25.2)	280 (29.1)	156 (27.1)
>55	24 (20.9)	275 (28.6)	125 (21.7)
Median age, y (IQR)	33 (25–52)	40 (24–57)	34 (22–53)
COVID-19 vaccines			
None	30 (26.1)	149 (15.5)	144 (25.0)
1	5 (4.3)	109 (11.3)	31 (5.4)
2	71 (61.7)	603 (62.7)	355 (61.7)
3	9 (7.8)	101 (10.5)	45 (7.8)
Mean (+SD)	1.5 (1.0)	1.7 (0.9)	1.5 (1.0)
Days since last vaccination, mean (+SD)	62.1 (54.3)	76.7 (44.7)	73.8 (40.4)
Sex			
F	78 (67.8)	658 (68.4)	408 (71.0)
M	37 (32.2)	304 (31.6)	167 (29.0)
Province			
Espaillat	60 (52.2)	309 (32.1)	203 (35.3)
San Pedro de Macorís	52 (45.2)	572 (59.5)	318 (55.3)
Santiago	3 (2.6)	81 (8.4)	54 (9.4)
Setting			
Rural	82 (71.3)	486 (50.5)	329 (57.2)
Urban	31 (27.0)	476 (49.5)	246 (42.8)
Seroprevalence, mean (+SD)	0.88 (0.33)	0.94 (0.25)	0.92 (0.27)
log_10_ spike antibody titer			
Mean (+SD)	2.4 (1.4)	2.6 (1.2)	2.5 (1.2)
Median (IQR)	2.6 (1.7–3.2)	2.7 (1.9–3.4)	2.6 (1.9–3.2)

*Values are no. (%) participants except as indicated. The study compared SARS-CoV-2 spike antibody data collected during July–October 2021 in the same Dominican Republic provinces from a longitudinal AFI surveillance system embedded in routine healthcare settings (“surveillance”) (*4*), which included routine blood collection for serologic testing; and a multistage, population-representative household serologic survey (“survey”) (*1*). Seroprevalence represents the percentage of participants with spike antibody titers above the manufacturer-specified cutoff (≥0.8 U/mL). IQR, interquartile range. †The matched survey dataset was generated through 1:5 nearest-neighbor propensity score matching of survey participants to surveillance participants, using age and number of COVID-19 vaccine doses as matching variables. Because only those variables were included in the propensity model, differences in other characteristics (e.g., province, setting, days since last vaccination) reflect inherent differences between the underlying sampling frames rather than confounding in the matched analysis. ‡Two surveillance participants were missing data for setting.

We included in the matched analysis all 115 surveillance participants and 575 matched survey counterparts (Table; Figure, panel A). We assessed seroprevalence, mean spike antibody levels, and the proportion of persons above thresholds corresponding to 75% protection against symptomatic SARS-CoV-2 infection (*5*). Unmatched mean antibody titers were similar: 2.4 log_10_ BAU/mL (95% CI 2.1–2.6) in the surveillance group versus 2.6 log_10_ BAU/mL (95% CI 2.5–2.7) in the survey group (p = 0.08 by 2-sample *t*-test). Results in the matched dataset were consistent (2.4 log_10_ BAU/mL in the surveillance group, 2.5 log_10_ BAU/mL in the survey group; p = 0.36). Matched seroprevalence was also comparable (surveillance: 88%; survey: 92%; p = 0.21 by 2-sample test for equality of proportions), and the percentage of persons with ≥75% protection differed by ≤6% (range 1.6%–5.4%) across all evaluated variants, with no statistically significant differences between groups (p = 0.18–0.97) (Figure, panels B, C; Appendix Table 1). Although the 95% CIs for the between-group differences in inferred protection slightly exceeded the prespecified +10-percentage-point margin for some variants, the observed differences were small (≤5 percentage points) and centered near zero, and the study had ≥80% power to detect differences of ≈6–8 percentage points, making large discrepancies between sampling frames unlikely.

**Figure:**
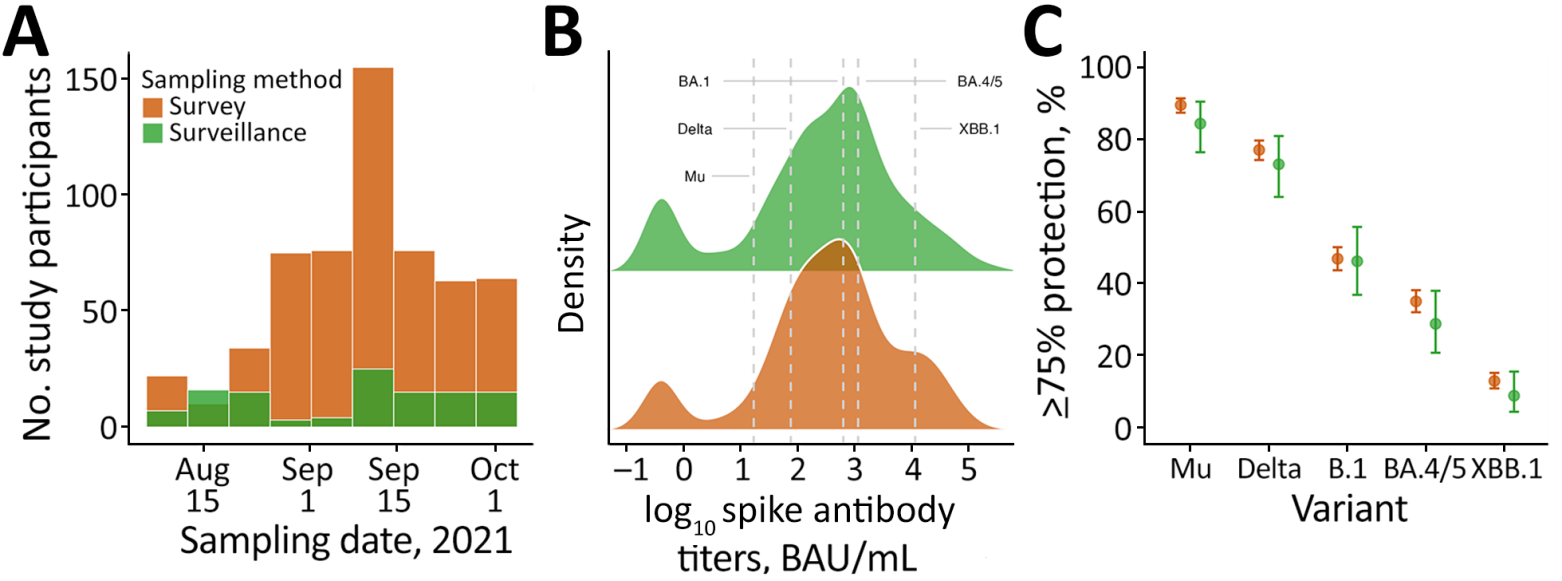
Spike antibody responses by surveillance and population survey sampling methods for study of acute febrile illness surveillance similar to household serosurvey for estimating population immunity, Dominican Republic. The study compared SARS-CoV-2 spike antibody data collected during July–October 2021 in the same provinces from a longitudinal AFI surveillance system embedded in routine healthcare settings (“surveillance”) (*4*), which included routine blood collection for serologic testing; and a multistage, population-representative household serologic survey (“survey”) (*1*). Participants were matched by age and number of COVID-19 vaccine doses at a 1:5 ratio (surveillance, n = 115; survey, n = 575). A) Histogram showing number of participants by sampling date. B) Density ridge plots illustrating titer distributions by sampling method. Dashed gray lines indicate previously reported spike antibody thresholds associated with >75% protection against symptomatic infection for Mu (10^1.23^), Delta (10^1.88^), BA.1 (10^2.80^), and BA.4/5 (10^3.06^). Threshold for XBB.1 was inferred based on ≈10-fold lower neutralizing response relative to BA.4/5. C) Dot-whisker plots showing estimated proportion of participants with antibody levels corresponding to >75% protection by variant or subvariant (underlying data in Appendix Table 1). Dots indicate point estimates; whiskers indicate 95% CIs. Protection thresholds taken from previously published variant-specific correlates of protection (*5*). Estimates show percentages of persons above those thresholds (uncertainty in thresholds [reported 95% CIs] not propagated into percentages).

Given their low cost, integration within routine care, and ability to sample broad community segments, AFI surveillance platforms may complement or substitute for traditional serosurveys when rapid situational awareness or repeated assessments are needed. Unlike cross-sectional serosurveys, this approach could also support ongoing monitoring over time (*4*,*5*), enabling near real-time tracking of antibody levels and inferred immunity and informing decisions about school reopening, travel restrictions, and vaccine targeting. Pooling data from multiple sites could further improve representativeness and reduce spatial or demographic bias. 

The primary limitation of this analysis is its cross-sectional design, which limits generalizability across timepoints. In addition, the study population had high exposure to SARS-CoV-2 vaccination and infection, which might not reflect settings with lower transmission and vaccination coverage (*6*). Nonetheless, our findings underscore the potential value of AFI and other clinical surveillance platforms as scalable, cost-effective tools for monitoring population immunity, particularly during pandemics, when speed, affordability, and feasibility are critical.

Appendix Additional information for acute febrile illness surveillance for estimating population immunity, Dominican Republic, 2021.
