# Supplementary material for: Acute Febrile Illness Surveillance for Estimating Population Immunity, Dominican Republic, 2021
Source: Emerg Infect Dis. 2026 Apr;32(4):660–3. doi: 10.3201/eid3204.251205 (PMC13094840; doi:10.3201/eid3204.251205)
Supplement: Appendix — Additional information for acute febrile illness surveillance for estimating population immunity, Dominican Republic, 2021. [file 25-1205-Techapp-s1.pdf]

Article DOI: http://doi.org/10.3201/eid3204.251205

EID cannot ensure accessibility for supplementary materials supplied by authors. Readers who have difficulty accessing supplementary content should contact the authors for assistance.

Acute Febrile Illness Surveillance for Estimating Population Immunity, Dominican Republic, 2021

Appendix

Methods

Setting, study design, participant selection, and ethical considerations: Two discrete studies were conducted in the Dominican Republic as part of a U.S. CDC-funded project (U01GH002238) that aimed to characterize the epidemiology of —and inform control of—acute febrile illnesses (AFI). The first consisted of prospective enrollment of symptomatic individuals aged ≥2 years presenting to two hospital-based AFI surveillance sites in the northwest and southeast of the country with undifferentiated fever (measured ≥38.0 °C or by history) or with new-onset anosmia or ageusia. The principal COVID-19 vaccines administered included the inactivated viral CoronaVac (Sinovac), the adenovirus vector ChAdOx1-S (Oxford/AstraZeneca) and mRNA BNT162b2 (Pfizer/BioNTech) vaccines. The study has been previously described (*1*). Nasopharyngeal swabs and venous blood were collected at enrollment. The second study was a national three-stage cross-sectional household cluster survey that enrolled asymptomatic individuals aged ≥5 years. The full sampling methodology for the national serosurvey has been described elsewhere (*2*). For both surveillance and survey participants, blood was processed into serum and stored at −80 °C. Written informed consent was obtained from all participants or their legal guardians. Adolescents aged 14–17 years provided written assent; children aged 7–13 years provided verbal assent. The studies were reviewed and approved by the National Council of Bioethics in Health (CONABIOS), Santo Domingo (013–2019); the Institutional Review Board of Pedro Henríquez Ureña National University, Santo Domingo; and the Mass General Brigham Human Research Committee, Boston, USA (2019P000094).

Immunoassay: Quantitative serum pan-immunoglobulin antibodies against the SARS-CoV-2 spike glycoprotein were measured at the Brigham and Women's Hospital (Boston, USA) using the Roche Elecsys SARS-CoV-2 electrochemiluminescence immunoassay (Roche Diagnostics, Indianapolis, USA), using a modified double-antigen sandwich format. Samples were considered reactive based on the manufacturer's cutoff index (≥0.8 U/mL). Values are reported as binding antibody units (BAU/mL), consistent with WHO standards. Spike antibodies measured with this assay have been demonstrated to inversely track with risk of symptomatic infection (*1*,*3*).

Virological assay: Acute SARS-CoV-2 infection was assessed using real-time reverse transcription polymerase chain reaction (RT-PCR) on nasopharyngeal specimens with the Allplex SARS-CoV-2 kit (Seegene, Seoul, South Korea), which targets the E, N, and RdRP genes. Amplification was performed under standard cycling conditions. Samples with a cycle threshold <37 were considered positive.

Data sources and processing: Serologic data from both the Surveillance and Survey datasets were restricted to participants sampled between August 7 and October 1, 2021—the only period during which both datasets overlapped in time and geography. The analysis was further limited to participants from the provinces of San Pedro de Macorís, Espaillat, and Santiago. In the Surveillance dataset, individuals with PCR-confirmed acute SARS-CoV-2 infection were excluded to minimize selection bias. As shown in our prior analyses (*3*), individuals with lower S-antibody titers are more susceptible to infection. Therefore, including PCR-positive participants would have selectively enriched the sample with individuals at the lower end of the antibody distribution. Otherwise, all individuals sampled during the specified period were included in both datasets. The Survey dataset was subsequently reduced from 962 to 575 participants through propensity score matching, as detailed below.

Propensity score matching: We performed 1:5 nearest-neighbor propensity score matching using Mahalanobis distance, adjusting for age and number of COVID-19 vaccine doses. We deliberately limited the matching variables to age and vaccination status to evaluate antibody distributions by sampling method using only routinely available data. This minimal-variable approach was intended to assess the feasibility of applying this method in settings where more detailed covariate data may not be accessible.

Estimation of protection proportions: We estimated the proportion of individuals with antibody levels corresponding to ≥75% protection against symptomatic SARS-CoV-2 infection using published immune correlates generated using the same Roche immunoassay for the following variants: Mu (1.23 $\log_{10}$ BAU/mL), Delta (1.88), B.1 (2.80), BA.4/5 (3.06) (*3*). The threshold for XBB.1 (4.06) was inferred based on a ~10-fold lower neutralizing response relative to BA.4/5. For each variant-specific threshold, we calculated group-wise proportions exceeding the cutoff, with 95% confidence intervals computed via exact binomial tests.

All analyses were conducted in R version 4.5.0 (2025–04–11), with visualization using *ggplot2*, and matching with *MatchIt*.

Appendix Table 1. Proportion of population with S-antibody titers above the level estimated to provide 75% protection against symptomatic SARS-CoV-2 infection*

Variant	Cutoff	Surveillance, % (95% CI)	Survey, % (95% CI)	Difference	P-value
Mu	1.23	84.3 (76.4, 90.5)	89.7 (87, 92.1)	5.4	0.188
Delta	1.88	73.0 (64.0, 80.9)	76.3 (72.7, 79.8)	3.3	0.510
BA.1	2.8	46.1 (36.8, 55.6)	43.3 (39.2, 47.5)	2.8	0.831
BA.4/5	3.06	28.7 (20.6, 37.9)	30.3 (26.5, 34.2)	1.6	0.969
XBB.1	4.06	8.7 (4.2, 15.4)	11.5 (9.0, 14.4)	2.8	0.774

*Estimates are based on a propensity-matched dataset including 575 Survey participants matched 5:1 by age and number of COVID-19 vaccine doses to 115 Surveillance participants. Proportions were calculated for each group, and 95% confidence intervals were derived using exact binomial methods. Differences in proportions between groups were assessed using two-sample tests for equality of proportions with continuity correction. Variant-specific cutoffs represent estimated immune correlates of protection for 75% vaccine efficacy: Mu (1.23 $\log_{10}$ BAU/mL), Delta (1.88), BA.1 (2.80), BA.4/5 (3.06), and XBB.1 (4.06, inferred from ≈10-fold reduced neutralizing activity relative to BA.4/5) (3).

Appendix Table 2. Number of COVID-19 vaccines received by age group among matched study participants*

Age group,			Survey		Surveillance	
y	Survey, N	Surveillance, N	One+ vaccine, %	Two+ vaccines, %	One+ vaccine, %	Two+ vaccines, %
0–14	55	10	0	0	0	0
15–34	239	52	78.2	71.1	76.9	71.2
35–54	156	29	85.9	76.9	79.3	72.4
55+	125	24	88	88	91.7	91.7

*Matching performed as detailed above by age and number of vaccines received. This table is intended to demonstrate the results of the matching on number of vaccine doses received by sampling method.

References

1. Nilles EJ, de St Aubin M, Dumas D, Duke W, Etienne MC, Abdalla G, et al. Monitoring temporal changes in SARS-CoV-2 spike antibody levels and variant-specific risk for infection, Dominican Republic, March 2021–August 2022. Emerg Infect Dis. 2023;29:723–33. PubMed https://doi.org/10.3201/eid2904.221628

2. Nilles EJ, Paulino CT, de St Aubin M, Restrepo AC, Mayfield H, Dumas D, et al. SARS-CoV-2 seroprevalence, cumulative infections, and immunity to symptomatic infection—a multistage national household survey and modelling study, Dominican Republic, June–October 2021. Lancet Reg Health Am. 2022;16:100390. PubMed https://doi.org/10.1016/j.lana.2022.100390

3. Nilles EJ, Paulino CT, de St Aubin M, Duke W, Jarolim P, Sanchez IM, et al. Tracking immune correlates of protection for emerging SARS-CoV-2 variants. Lancet Infect Dis. 2023;23:153–4. PubMed https://doi.org/10.1016/S1473-3099(23)00001-4
